# Associations between Mild Behavioral Impairment and Functional Impairment in Cognitively Unimpaired Community‐dwelling Older Latinos

**DOI:** 10.1002/alz70857_107546

**Published:** 2025-12-26

**Authors:** Jorge Alcina, Nikole A Bonillas Félix, Randy Medrano, Lusiana Martinez, Alexandre Closs Fanfa Ribas, Nadeshka J Ramirez‐Perez, Averi Giudicessi, Jairo E. Martinez, Isabel Solis, Catarina Tristão‐Pereira, Vincent Malotaux, Bing He, Liliana Ramirez Gomez, Jennifer R. Gatchel, Marta Gonzalez Catalan, Clara Vila‐Castelar, Daniel G Saldana, Yakeel T. Quiroz

**Affiliations:** ^1^ Massachusetts General Hospital, Harvard Medical School, Boston, MA, USA; ^2^ McLean Hospital, Harvard Medical School, Belmont, MA, USA; ^3^ Department of Psychological & Brain Sciences, Boston University, Boston, MA, USA

## Abstract

**Background:**

Latinos are 1.5 times more likely than non‐Latino Whites to be diagnosed with Alzheimer's Disease and Related Dementias (ADRD). Mild Behavioral Impairment (MBI), characterized by new neuropsychiatric symptoms in adults over 50, is associated with cognitive impairment, dementia, and increased functional burden in later life. However, the relationship between MBI and cognition remains unexplored in Latinos. This study examined the effects of self‐reported MBI severity and functional impairment in cognitively‐unimpaired community‐dwelling older Latinos.

**Method:**

126 cognitively‐unimpaired Spanish‐ and/or Portuguese‐speaking participants from the Boston Latino Aging Study (BLAST) were included, ages 55 years and older, 80.3% female, mean age 67.07 ±  7.73 years, mean education 11.69 ±  4.63 years. Neuropsychiatric symptoms were measured by the Mild Behavioral Impairment Checklist (MBI‐C; self‐report). Two out of five MBI‐C domains, namely the interest motivation and drive (IMD) and mood anxiety (MA) domains were investigated, with higher scores denoting greater behavioral difficulties. Functional impairment was measured with the Functional Activities Questionnaire (FAQ; informant‐report), with higher scores denoting greater functional difficulties. Depressive symptoms were measured using the Geriatric Depression Scale (GDS). Global cognition was measured through the Mini‐Mental State Examination (MMSE). Stepwise linear regression models examined the effect of MBI‐C domains on functional impairment, adjusting the models for age, sex, education, and global cognition.

**Result:**

Mean total MBI‐C IMD domain score was 1.88 ±  2.93, mean total MBI‐C MA domain score was 2.28 ±  3.13, and mean total FAQ score was 3.25 ±  5.65. A minority of participants (20%) reported clinically‐significant depressive symptoms (GDS ≥ 10). Higher MBI‐C IMD scores (*β* = .517, *p* < .001), but not MBI‐C MA scores (*β* = .119 *p* > .05), were associated with higher FAQ scores (*F* [4, 53] = 9.366, *p* < .001, *R^2^_adjusted_* = .370).

**Conclusion:**

Cognitively unimpaired community‐dwelling older Latinos reported symptoms of decreased motivation that were associated with greater functional burden in daily living skills. Our findings highlight the need to assess and manage neuropsychiatric symptoms among Latino older adults. Future longitudinal studies with larger samples are needed to further understand functional effects of neuropsychiatric symptoms in Latinos and other minoritized groups.

